# Postcataract surgery outcome in a series of infants and children with Down syndrome

**DOI:** 10.1136/bjo.2007.134619

**Published:** 2008-07-24

**Authors:** C Gardiner, B Lanigan, M O’Keefe

**Affiliations:** The Children’s University Hospital, Temple Street, Dublin 1, Ireland

## Abstract

**Aims:**

To report the visual and refractive outcome and complications in children with Down syndrome undergoing cataract extraction.

**Methods:**

The case notes of 18 infants and children with lens opacities and Down syndrome who underwent cataract extraction between January 1981 and August 2006 were reviewed.

**Results:**

Over the 25-year study period, 7% (33 eyes) of paediatric eyes undergoing cataract extraction had Down syndrome. The average follow-up time was 11.2 (SD 7.5) years with a range of 2.5 months to 25 years. 25 were congenital, and eight were developmental lens opacities. 40% of patients attained a postoperative BCVA between 6/9 and 6/18. There was a large myopic shift of −7.96 (4.7) D for aphakes and −8.06 (7.4) D for pseudophakes with an average increase in axial length of 3.58 (3.14) mm. There was a 30% incidence of posterior capsular opacification (PCO) overall, 38% in eyes without a primary posterior capsulotomy. Five eyes developed aphakic glaucoma, one eventually necessitating an enucleation. Two patients had retinal detachments on follow-up.

**Conclusion:**

Cataract extraction in our population of children with Down syndrome is a safe and effective procedure with a very encouraging visual outcome.

Down syndrome is the most common genetic cause of developmental disability in Ireland with a prevalence of one in 546 live births,^1^ the highest in Europe. An Irish cross-sectional study of 394 children and adolescents with Down syndrome found that 50% of the children had ophthalmic pathology, high myopia most commonly (24.9%) with 4.6% having cataracts.^3^

There is an increased incidence of both acquired (11–60%^2^ ^4^^–^6) and congenital cataracts (2–6%^6^ ^7^ ^11^ ^12^).

This retrospective study reports the visual and refractive outcome and complications in children with Down syndrome undergoing cataract extraction.

## PATIENTS AND METHODS

Thirty-three eyes of 18 infants and children with Down syndrome and cataracts underwent surgery with follow-up between January 1981 and August 2006. Ophthalmological data were recorded from medical records of assessments performed prior to surgery and at follow-up.

Pre-, initial and final postoperative vision were assessed using the appropriate method for the age and capability of the child. Change in refractive error, as well as keratometric and axial length measurements, when performed, was obtained from follow-up EUAs.

Consecutive surgery was performed for bilateral cataracts. The surgical technique evolved from pars plana lensectomy to an anterior segment approach using either a limbal based incision with can opener capsulotomy or a scleral tunnel approach with a continuous curvilinear or diathermy capsulorhexis. The diathermy needle was also used to create a posterior capsulorhexis with or without an anterior vitrectomy in later cases.

Placement of an intraocular lens (IOL) was recorded, and the appropriate power was calculated with the SRK II formula—in general those less than 1 year were made 6 D hyperopic, between 1 and 2 years 4 D hyperopic, between 2 and 6 years 2 D hyperopic and greater than 6 years emmetropic.

Postoperatively congenital cataract cases received intravenous methylprednisolone 2 mg/kg/day. Intraoperatively, all had a subconjunctival injection of dexamethasone 2 mg and an antibiotic, and in later patients intracameral dexamethasone 400 μg was administered.

The study period was 25 years. The average follow-up time was 11.2 (7.5) years with a range of 2.5 months to 25 years. Long-term complications such as glaucoma, posterior capsular opacification (PCO), nystagmus and strabismus were recorded.

## RESULTS

During the 25-year study period 1981–2006, a total of 467 eyes underwent cataract extraction for paediatric lens opacities. Of these, 33 (7%) had Down syndrome, 12 females and six males.

There were 25 eyes with congenital cataract. Eleven patients had bilateral cataracts, and three had unilateral cataracts. Developmental cataracts, which were all bilateral, were diagnosed in four children. The median age at surgery was 5 weeks (range 2 weeks to 33 months) for congenital cataracts and 8.1 years (97.5 months, range 4–11.6 years) for those with developmental lens opacities.

One patient had persistent fetal vasculature (PFV).

There were nine aphakes (16 eyes) and nine (17 eyes) pseudophakes with primary PCIOLs. Of the latter, 11 had congenital, and six had developmental cataracts. Eight eyes had one-piece heparin coated polymethyl methacrylate (PMMA) lenses, and nine had foldable acrylic implants.

Preoperative visual acuities were documented using visual function patterns due to early age at presentation and the diagnosis of Down syndrome, which made more formal visual assessment very difficult. The initial postoperative visual acuity was not documented in five patients due to poor cooperation, but all were able to carry out subsequent visual assessments (see tables 1, 2).

**Table 1:** Patient characterisitics

Patient no n = 18	Sex	Type of cataract UL or BL	Age at surgery	IOL implantation	(i) PPC	(i) PCO	Glaucoma	Other complications
					(ii) Anterior vitrectomy	(ii) Yag PC		
						(iii) Surgical PC		
1	F	UL congenital “cortical’	5/52	No	(i) No	(i) Yes	No	Keratitis
					(ii) No	(ii) Yes×1		
2	F	BL “dense central” develop mental	4 years	No	(i) No	(i) No	No	No
					(ii) No			
3	F	BL “dense mature” congenital	5/52	Yes	(i) No	(i) Yes BL	No	Convergent
					(ii) No	(ii) Yes×2		Strabismus
						(iii) Yes×2		
4	F	BL “dense” congenital	R 4/52	Yes	(i) Yes	No	No	Exotropia
			L 2 years 2/12		(ii) Yes			
5	F	BL develop mental “cortical and nuclear’	11 year 6/12	Yes	(i) No	(i) Yes L	No	No
					(ii) No	(ii) Yes		
6	F	BL “central” congenital	2/52	No	(i) Yes	(i) No	No	Convergent strabismus
					(ii) No			× 2 strabismus repairs
7	M	BL “dense” Congenital	3/52	Yes	(i) No	(i) Yes BL	No	Anterior
					(ii) No	(ii) Yes×2		Synechiolysis at 10/12
						(iii) Yes×1		
8	M	BL “lamellar” congenital	23/12	Yes	(i) Yes	(i) Yes UL	No	Convergent
					(ii) No	(ii) Yes		Strabismus operated on
9	M	BL “central anterior polar capsular” developmental	10 years	Yes	(i) No	(i) No	No	Zonular
			1/12		(ii) No			Rupture
10	F	Congenital	10/12	Yes	(i) Yes	(i) No	No	Exotropia
		BL			(ii) No			
11	F	BL “cortical and nuclear” developmental	6 years 2/12	Yes	(i) No	(i) Yes BL	No	Esotropia
					(ii) No	(ii) Yes		
12	F	Congenital	3/52	Yes	(i) Yes	No	No	No
		UL			(ii) Yes			
13	M	Congenital	3/52	No	(i) No	No	Yes	Removal of lens matter
		BL			(ii) Yes			Stitch abscess
								Aphakic glaucoma
14	F	Congenital	Soon after birth early 1980s not documented	No	(i) No	(i) Yes	Yes	Iris prolapse postop with wound repair and anterior vitrectomy
		BL			(ii) No	No treatment		Right trabeculectomy ×2 left broad iridectomy and division of anterior synechiae
								Right buphthalmos
								Right enucleation
15	F	BL “dense” congenital	3/52	No	(i) No	No	No	Right retinal
					(ii) No			Detachment 1992
								Esotropia
16	F	BL “dense lamellar” Congenital	4/12	No	(i) No	No	No	Convergent
					(ii) No			Strabismus
17	M	Congenital	2 years 9/12	No	(i) No	No	Yes	Keratitis
		BL			(ii) No			R buphthalmos
								R retinal detachment
18	M	UL congenital PFV	6/52	No	(i) No	PFV	No	Microphthalmos
					(ii) No			PFV

BL, bilateral; IOL, intraocular lens; L, left eye; PCO, posterior capsular opacification; PFV, persistent fetal vasculature; PL, perception of light; PPC, primary posterior capsulotomy; R, right eye; UL, unilateral.

**Table 2:** Refractive and visual outcome

Patient no	Initial postoperative refractive error (D)	Last postoperative refractive error (D)	Change in refractive error	Initial postoperative visual acuity	Final postoperative visual acuity
Aphakic postoperative refractive error and visual acuity					
1	+20	+10.25	−9.75	Not documented	6/14 FCPL BEO
					6/36 FCPL R
					6/36 FCPL L
2	+10.5	+9.5	−1.0	Not documented	CSM followed up in another unit
	+12	+9.5	−2.5	Not documented	CSM followed up in another unit
6	+15.5	+4.5	−11.0	6/24 BEO Kays pictures	6/19 Snellen
	+13.25	+7.0	−6.25		6/24+2 Snellen
13	+19	+8	−11	6/18 SG	6/36 Allen
	+19	+8	−11	6/18 SG	6/12 Allen
14	AE				
	+8.25	+6.0	−1.75	CUSUM	?6/12 SG
15	+16	+4	−12.0	CUSUM	PL
	+15	+3.5	−11.5		6/18 Kays pictures
16	+20	+7	−13	Not documented	CUSM
	+20	+7	−13	Not documented	CUSM
17	+8.5	+3	−5.5	Not documented	F+F
	Buphthalmos			Not documented	PL
18	Microphthalmia				UCUSUM PHPV
Pseudophakic postoperative refractive change and visual acuity					
3	+8	+1.25	−6.75	3/60 BEO FCPL	6/18 Kays pictures
	+8	+0.50	−7.50		6/18 Kays pictures
4	+4	−9.5	−13.5	F+F	6/9 Kays pictures
	+0.50	−3.5	−4	F+F	6/9 Kays pictures
5	+2	+0.50	−1.5	6/12 logMAR	6/9 SG
	+2	+1.00	−1.0	6/12 logMAR	6/15 SG
7	+4	−19	−23	CSM	3/6 Kays pictures
	+5.75	−18	−24	CSM	3/12 Kays pictures
8	+2.25	−8	−10.25	6/24 BEO FCPL	6/30 FCPL
	+1	−8	−9		6/30 FCPL
9	−0.25	+0.75	+1.00	Not documented	CSM
	0	1	+1.00	Not documented	CSM
10	+2.5	−5.25	−7.75	Fixing and following BE	6/12 Kays pictures
	+2.25	−3.75	−6		6/12 Kays pictures
11	+2.5	+1.00	−1.5	6/12 Kays pictures	6/18 SG
	+2.5	+1.75	−0.75	6/12 Kays pictures	6/18 SG
12	+7.5	−4.00	−11.50	6/130 FCPL	6/12 Kays pictures

AE, artificial eye; BEO, both eyes open; CSM, central steady and maintained; CUSM, central unsteady and maintained; CUSUM, central unsteady and unmaintained; D, dioptres; FCPL, forced choice preferential looking; L, left eye; PL, perception of light; R, right eye; SG, Sheridan Gardiner; UCUSUM, uncentral unsteady and unmaintained.

Regarding the final postoperative visual acuity, six (10 eyes) patients could not cooperate or had vision which was too poor for a formal vision assessment. Of these, two had central steady and maintained (CSM) vision bilaterally by the fixation method, and one had central unsteady and maintained (CUSM) vision bilaterally in association with a convergent strabismus. Two patients had “perception of light” (PL) and “hand movements” (HM) vision secondary to retinal detachments, but the fellow eye in both of these patients was better with one fixing and following (F+F) and the other achieving 6/24 with Kays pictures. One patient had uncentral unsteady and unmaintained (UCUSUM) vision, but this patient had coexisting PFV. Five of the six were aphakes.

One patient underwent an enucleation for buphthalmos and is currently wearing a prosthesis.

The remaining 22 eyes—seven aphakic and 15 pseudophakic—were able to perform forced choice preferential looking (FCPL), Kays pictures, logMAR, Sheridan Gardiner (SG), Cardiff or Allen Cards acuity testing.

Thirteen eyes (40% overall and 60% of those able to perform formal visual assessments)—11 with congenital and four with developmental lens opacities—had attained a level of 6/9–6/18 vision by whatever method at final review.

Of the remaining nine eyes, two patients attained 6/24 vision with Kay pictures in one eye, three patients achieved 6/36, one with Snellen visual acuity testing in either eye and two with FCPL and Sheridan Gardiner testing in one eye, one had 6/30 in each eye with FCPL, and one had 6/60 with Allen cards in one eye.

The average initial aphakic refractive error postoperatively was +14.4 (SD 5.06) D. The average postoperative error at final review was +6.4 (2.3) D. This is an average myopic shift of −7.96 (4.7) D.

The corresponding average initial pseudophakic refractive error postoperatively was +3.20 (2.6) D. The final refractive error averaged at −4.85 (6.5) D thus an average myopic shift of −8.06 (−7.4) D (table 2).

The mean refractive shift of those with congenital cataracts alone operated on within the first 12–24 months of life was −11.5 (5.2) D (initial average +10.6 (7) D and final average −0.7 (8.7) D.

Axial length data at the time of surgery was available on 19 pseudophakic eyes. The average axial length was 20.62 (2.56) mm with a range of 16.38–23.83 mm. Initial and follow-up axial lengths were available on 11 eyes. The average initial axial length of these patients was +19.58 (2.6) mm, and the average axial length at follow-up EUA was 23.16 (2.56) mm. This is a change of 3.58 (3.14) mm over an average follow-up period of 74.67 months (6.2 years). All were in the congenital cataract group.

During follow-up, 30% of eyes (10 eyes, seven patients) developed PCO. Two of the patients were aphakic, and five were pseudophakic.

Five eyes (50%) with PCO were noted within 6 weeks postoperatively. The other five eyes had PCO at 8 months’, 22 months’, 2 years’, 3 years’ and 10 years’ follow-up, respectively. The median age at which cataract surgery was carried out in these eyes was 12 months (range 3 weeks to 11 years).

There was a 38% (nine of 24) incidence of PCO in eyes with no primary posterior capsulotomy (PPC). Three were noted at follow-up outpatient examination at 3 weeks, then at 5 weeks, 6 weeks, 8 months, 22 months, 2 years and 10 years, the last not requiring treatment. All the other eight eyes (89%) underwent Yag capsulotomies, and two of these necessitated additional surgical capsulotomies.

Of the nine eyes (seven pseudophakic and one aphakic) that underwent posterior capsulotomy, only one (pseudophakic) (11%) developed PCO at 3 years’ follow-up and underwent Yag capsulotomy for this.

There were five eyes with aphakic glaucoma noted at 2 weeks, 2 months, 5 months and 9 years. Three underwent trabeculectomies. One patient underwent bilateral trabeculectomies, repeated with antimetabolite application, repeated cyclodiode treatments bilaterally and finally bilateral Ahmed valve insertion, one requiring enhancement and the other shortening before control was reached in conjunction with topical antiglaucomatous treatment. One blind eye was referred with severe buphthalmos following repeated trabeculectomies with macular scarring, and this eye underwent an enucleation. This patient’s other eye has also recently developed glaucoma maintained on topical treatment. The fifth eye, which had buphthalmos and keratoconus with a scarred vascularised cornea and poor vision, underwent repeated cyclocryotherapy to achieve control. There were no cases of pseudophakic glaucoma.

Seven patients had esotropia, and two had exotropia. Three of the esotropes underwent strabismus repair procedures.

Two patients had retinal detachments on follow-up. The patient mentioned above who was blind with buphthalmos, aphakic glaucoma and a scarred keratoconic cornea had a retinal detachment detected by ultrasound during a routine EUA 25 years after removal of his congenital cataracts. As this patient had no visual potential, the retinal detachment was not operated on. The other patient who had congenital cataracts diagnosed at birth and underwent bilateral lensectomies in 1989 then developed a rhegmatogenous macula off retinal detachment 3 years later. She underwent successful retinal detachment surgery, but her vision remains at HM.

## DISCUSSION

The association between lens opacities and Down syndrome was first described in 1910 by Pearce *et al*,^9^ and various varieties of cataract have been described in the literature.^7^ ^8^ ^10^ However, although flake opacities are the most common, there appears to be no distinctive type of lens opacity in this condition.^7^

A 4.6% incidence of cataract in children between 3 months and 19 years old with Down syndrome in Ireland has recently been reported.^3^ A recent population-based study of Down syndrome and early cataract (congenital and developmental from 0 to 17 years) from Denmark, however, found a frequency of only 1.4%, with only 1% being optically significant.^12^

Our study found an incidence of Down syndrome in 7% of eyes (33 eyes of 467) undergoing surgery for lens opacities over the study period.

Other studies show 5.4% (13 patients of 243) in the UK^11^ and 2.8% (29 patients of 1027) in Denmark^12^ of patients with early cataract in general to also have Down syndrome.

Congenital lens opacities in Down syndrome are rare; indeed some authors question the need for screening at all.^2^ Reported prevalences vary as often only “those cataracts which would seem dense enough to cause visual difficulty”^4^ ^8^ are reported.

However, in our series, 76% (25 eyes of 33) of the eyes proceeding to cataract extraction were congenital lens opacities, which may reflect the early aggressive surgical intervention in our centre combined with the meticulous follow-up of infants with early cataract. Of these 25 eyes, the majority (22 eyes) also underwent surgery within 1 year.

Of the three eyes with congenital lens opacities that had surgery after 1 year, one child was diagnosed at birth with a dense right-sided lens opacity with no fundal view; the left lens was clear at this stage. She underwent a right cataract extraction 4 weeks later but, at follow-up 2 months later, was noted to have developed a left-sided posterior subcapsular cataract which was observed for two further years until it was removed. The second child was first seen and diagnosed as having bilateral congenital cataracts at 1 year. These were initially treated with mydriatic (atropine), as there was a good red reflex and patching before proceeding to right cataract extraction at 2 years and 9 months and left at 3 years.

Another possible explanation for this high incidence of congenital lens opacities is that the older children with Down syndrome may have been seen at other centres and undergone surgery elsewhere. Our findings are therefore at variance with some other studies, as a dramatic increase after puberty has been described with higher incidences of up to 55% or more of Down syndrome children with developmental cataracts after 10 years.^4^ ^6^ ^7^ ^9^ ^10^ Jaeger found 13% with some form of cataract between 12 and 22 years,^5^ Pires da Cunha and Moriera also found cataracts in the same percentage of their group of patients with a significantly more frequent occurrence in patients 12 years of age and older versus below 11 years of age.^6^

Studies of visual and surgical outcome in this group of patients are limited.

Cullen in 1963 noted that “these eyes react unfavorably to intraocular surgery.”^13^ Hiles *et al* aspirated 14 mature cataracts—six within the first year of life, in eight children with Down syndrome. Although three eyes were lost by self-mutilation, there was a visual improvement in the other cases with an enhanced awareness of surroundings.^14^

In Haargard and Fledelius^12^ recent study, 14 patients (26 eyes) out of 56 eyes with cataract and Down syndrome underwent cataract surgery at a median age of 2 years, but only three cases had primary IOLs implanted. This is compared with 17 of 33 eyes operated on in our study. The authors proceeded to cataract surgery in 11 eyes of six patients within the first year of life. In our study, 22 patients of 24 eyes with congenital cataracts underwent cataract extraction before the age of 1 year. The remaining two were operated on at the age of 2, one for a congenital cataract which had progressed from originally being insignificant and the other for a case that had been treated for amblyopia with patching and mydriatics for some time preoperatively.

Our visual outcome was very encouraging with 40% overall and 60% of those (aphakes and pseudophakes) who were able to carry out a formal visual assessment attaining a level of 6/9–6/18 vision. There is no comparable study on visual outcome in this group of patients in the literature.

In general in aphakic eyes, there is a myopic shift of up to 10 D from infancy to adulthood, most of which occurs in the first year of life. This is much larger than the average of 0.9 D in normal phakic children.^15^ ^16^ Long-term pseudophakic myopic shifts of 6.6 D,^16^ 3.26 D (congenital) and 0.96 D (developmental),^17^ and 6D^18^ have been published. The last figure, reported by O’Keefe *et al*, occurred most often in the first 24 months with a bigger myopic shift in children with Down syndrome.^18^

This is also seen in our study where we found a large average myopic shift of −7.96 (4.7) D for aphakic eyes and a corresponding large average pseudophakic myopic shift of −8.06 (7.4) D. This was also seen to a greater extent in those with congenital cataracts operated on within the first 12–24 months of life (−11.5 (5.2) D).

An increase in axial length of 3.58 (3.14) mm over 6 years was noted for 11 pseudophakic eyes in our study. All these were in the congenital cataract group.

Similarly, Flitcroft *et al* reported a mean increase of 3.41 mm over 140 weeks.^17^

We had a 30% incidence of posterior capsular opacification documented overall in aphakes and pseudophakes, most of the cases occurring in the congenital group (see results) with a median age at which surgery was carried out of 12 months.

The prevalence of secondary membranes in general in aphakic patients in the literature varies between 5 and 13%.^19^^–^21 Zwaan *et al* had a 39% incidence of PCO in his paediatric pseudophakic population, with Gimbel’s group necessitating a secondary Yag capsulotomy in 47.9%.^22^ ^23^ Fifty per cent of bilateral cases required surgical capsulotomies in Haargaard’s series with Down syndrome.We previously reported an incidence of 50% requiring posterior capsulotomy for pseudophakes with developmental cataract and 83.3% for congenital pseudophakes in eyes with no PPC. This was reduced to 20% and 33.3% with a PPC.^24^

There were five cases of aphakic glaucoma (44% of aphakes) and no cases of pseudophakic glaucoma to date in this report. Haargaard reports one case of three pseudophakes with Down syndrome having glaucoma but does not comment on the incidence in the aphakes. Comparing with the general literature, Levin and Simon report incidences of aphakic glaucoma in 22–24% of their patients who underwent surgery for congenital cataracts consistent with other reports.^19^–^21^ ^25^

Nine (50%) patients had strabismus, with three undergoing strabismus repair procedures—similar to Haargaard’s (51%)^12^ and Levin’s (52%)^21^ series.

Two (6% of eyes) patients had retinal detachments, 3 and 25 years postoperatively. In Haargaard’s series, five eyes developed retinal detachments postoperatively after prolonged latencies.

The incidence of retinal detachment in the literature in general following surgery for congenital cataract has been estimated at between 1%^26^ and 1.5%,^27^ but while our rate of postoperative detachment is higher than this (6%), the long period of follow-up in our study group should also be taken into account.

## CONCLUSION

As there is a higher rate of congenital and developmental cataract in Down syndrome when compared with the general population, management of visually significant cataract is of prime importance in this group.

Patients with Down syndrome are also considered ideally suited to IOL implantation because of difficulties with contact lenses or spectacle wear.

This study has shown that cataract extraction with or without IOL implantation is an effective and worthwhile procedure with a good visual outcome. The large myopic shift in this cohort of patients has previously been described and must be taken into account when planning surgery.
